# The Rhinolith—A Possible Differential Diagnosis of a Unilateral Nasal Obstruction

**DOI:** 10.1155/2010/845671

**Published:** 2010-06-17

**Authors:** Detlef Brehmer, Randolf Riemann

**Affiliations:** ^1^Private ENT Clinic Goettingen, Faculty of Medicine, University Witten/Herdecke, Friedrichstraße 3/4, 37073 Goettingen, Germany; ^2^Department of Otorhinolaryngology, City Hospital Frankfurt-Hoechst, Gotenstr., 6–8, 65929 Frankfurt, Germany

## Abstract

Rhinoliths are mineralised foreign bodies in the nasal cavity that are a chance finding at anterior rhinoscopy. Undiscovered, they grow appreciably in size and can cause a foul-smelling nasal discharge and breathing problems. Giant nasal stones are now a very rare occurrence, since improved diagnostic techniques, such as endoscopic/microscopic rhinoscopy, now make it possible to identify foreign bodies at an early stage of development. We report the case of a 37-year-old patient who, at the age of 5-6 years, introduced a foreign body, probably a stone, into his right nasal cavity. On presentation, he complained of difficulty in breathing through the right nostril that had persisted for the last 10 years. For the past four years a strong fetid smell from the nose had been apparent to those in his vicinity. Under general anaesthesia, the stone was removed in toto from the right nasal cavity. The possible genesis of the rhinolith is discussed, our case compared with those described in the literature, and possible differential diagnoses are considered.

## 1. Introduction

Today, rhinoliths are a rare occurrence. Rhinoliths are mineralised foreign bodies in the nasal cavity that are a chance finding at anterior rhinoscopy. The foreign body finds its way into the nasal cavity almost always through the limen nasi. According to Denker and Brünings [1], such a situation was formerly most commonly observed in children and the mentally retarded, who “for a lark,” as it were, inserted such small objects as beads, small stones, coins, and suchlike into a nostril. Trauma, surgical operations and dental work, nasal packaging material, and plugs of ointment may also promote the development of a rhinolith. In addition, vomit may enter the nose via the choana and remain there forming a foreign body. Finally, a rhinolith may develop spontaneously, for example in the case of a long-standing chronic polypoid sinusitis with accumulation of secretions followed by mineral deposition [2, 3]. Provided that the endonasal mucosa is intact, any tiny particles that may enter the nose during inspiration are eliminated through the secretion of mucus and ciliary action. If the mucosa is damaged, such particles may remain in the nasal cavity and grow in size through accretion of mineral salts and incrustation. As the rhinolith increases in size, the symptoms to which it gives rise may range from unilateral nasal discharge, unilateral purulent rhinitis with or without consecutive sinusitis, facial pain, headache, epistaxis, impairment of nasal breathing ending in complete obstruction, dacryocystitis, otorrhea [4], foetor, anosmia, palatal perforation [3, 5], and septal perforation [6]. The duration of the medical history may range from months to decades [7], and women appear to be more commonly affected than men [8]. Although most rhinoliths are detected in young adults, they may be found at any age (6 months to 86 years) [5, 9, 10]. The diagnosis is established on the basis of the medical history and endoscopic findings; an imaging modality may provide additional information.

## 2. Case Report

**Figure 1:**
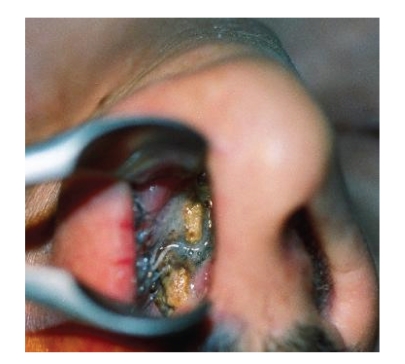
Rhinolith in the right nasal cavity.

**Figure 2:**
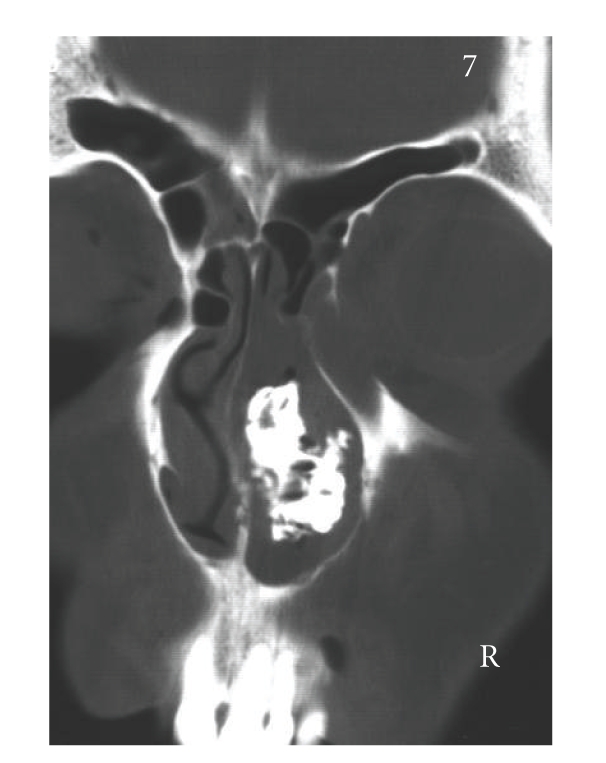
Coronal CT showing a calcified space-consuming lesion occupying much of the right nasal cavity.

**Figure 3:**
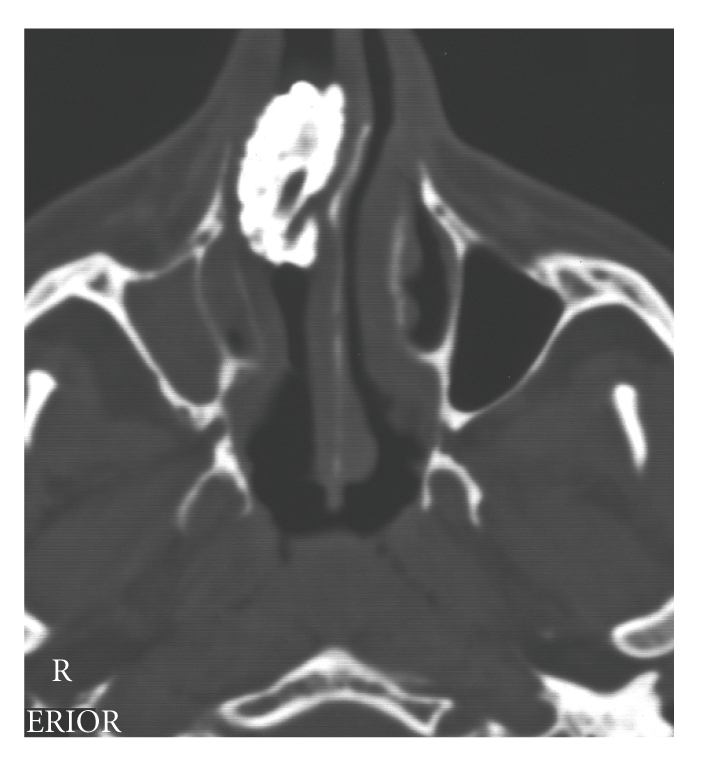
Axial CT scan showing the rhinolith in the right nasal cavity, with consecutive shadowing of the right maxillary sinus.

**Figure 4:**
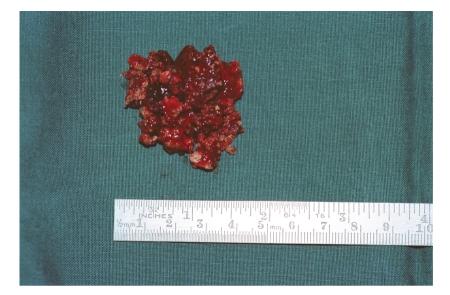
Removed rhinolith.

According to his own recollection, the 37-year-old patient had, at the age of 5 or 6 years, inserted a stone into his right nasal cavity. Over the course of time, this event had been completely forgotten. He now presented, with right nasal obstruction accompanied by a purulent discharge from the right nostril and a foul smell from the nose, of which he himself was unaware. After clearing the nasal cavity of the secretion by aspiration, and detumescence of the mucosa, a blackish solid foreign body was detected at the level of the piriform aperture, which almost completely occluded the right nasal cavity (Figure 1). The axial/coronal CT scan of the nasal cavity, obtained to exclude bony destruction, revealed a large, dense, space-consuming lesion measuring between one and a maximum of three cm in diameter located in the inferior and middle meatus on the right, and presenting partly regular, partly irregular margins and caused shadowing of the right maxillary sinus (Figures 2 and 3). No bony destruction was evident. Under general anaesthesia, the rhinolith was broken into two fragments and removed (Figure 4). In addition, the right maxillary sinus was cleared out via an infraturbinal window. After 32 years in situ, the foreign body had displaced the intact septum to the left. The inferior and middle turbinates were atrophic. Histological examination of the biopsy material excised from the mucosa of the nasal cavity and septal mucosa revealed chronic, florid, ulcerous, nonspecific, in part hyperplastic, and polypoid inflammation. After applying the usual postoperative care, the patient became symptom-free, and an endoscopic inspection of the maxillary sinus performed on the 5th postoperative day revealed healed, bland endothelial mucosa.

## 3. Discussion

The first published report of a calcified foreign body in the nose appeared in 1654, in which Bartholini described a stone-hard foreign body that had grown around a cherry stone [11]. The term rhinolith was first coined in 1845 to describe a partially or completely encrusted foreign body in the nose [11]. Calcified incrustations in the nasal cavity were subjected to a chemical analysis, first by Axmann in 1829 [12], and thereafter by various other authors [2, 11, 13–17]. In general they comprise 90% inorganic material, with the remaining 10% being made up of organic substances incorporated into the lesion from nasal secretions. Mineralogical investigations employing powder diffractometry unequivocally identified the mineral whitlockite (Ca_3_  (PO_4_)_2_)  as representing the main constituent of a rhinolith. In addition, the mineral apatite (Ca_5_  (OH, F, CI)  (PO_4_)_3_) and carbonated apatite (dahlite) have also been identified. Another author describes an extremely rare iron-containing rhinolith, the X-ray diffraction analysis of which revealed siderite (Fe^2+^  CO_3_ and ferrihydrite (5Fe_2_O_3_ × 9  H_2_O) [18]. This author suspected an exogenous iron-containing nidus to be the likely cause, since the endogenous development of an iron-rich rhinolith is not conceivable; the physiological secretions (nasal mucus, tears) produced in the nose contain no demonstrable amounts of iron.

The calcified foreign bodies in the nose were formerly designated false or true rhinoliths. Today, these terms have been replaced by exogenous and endogenous, depending on whether or not a nucleus, around which the incrustation has been deposited, can be found. Those rhinoliths that have developed around nonhuman material introduced into the nose and remaining in situ such as cherry stones, stones, forgotten nasal swabs, or similar objects are termed exogenous. Endogenous rhinoliths are those that have developed around the body's own material such as, for example, ectopic teeth in the maxillary sinus, bone sequesters, dried blood clots in the nasal cavity, and inspissated mucus [19, 20]. Some 20% of the rhinoliths are of endogenous origin [19]. The pathogenesis of rhinolith development has still not been completely elucidated. The following four conditions for the development of such a lesion are generally accepted and recognised.

1. The foreign body introduced into the nose must give rise to an acute or chronic inflammation of the nasal mucosa with consecutive suppuration.
2. The putrid discharge must have a high content of calcium and/or magnesium.
3. The mechanical obstruction must block the outflow of pus and mucus.
4. The secretion must be exposed to a current of air, to concentrate pus and mucus and permit the mineral salts to precipitate, and thus give rise to Incrustation.

The last point is presumably the reason for the fact that an antrolith in the maxillary sinus is only a rare occurrence [19]. To date, there have been no reports of a calcified foreign body in any of the other sinuses. Rhinoliths almost always occur unilaterally. Kharoubi [21] reported an unusual case of bilateral rhinolithiasis subsequent to destruction of the posterior nasal septum.

Time is a major factor in the development of a rhinolith. The literature contains information on different in-situ durations [2, 10, 14, 21]. One author describes the case of a woman in whom, a sharp irrigation of the maxillary sinus was performed at the age of ten, absorbent cotton wool had been introduced into the nose and forgotten. 27 years later, she attended an ENT clinic complaining of impaired nasal breathing. Following an inspection of her nose she was informed that her breathing was “normal,” and an operative exploration was not done. On account of the foul smell from her nose, the patient was socially isolated and never married. Some 8 years later, her persistent breathing problem prompted her to make a further attempt to have it surgically treated. Once again the rhinolith remained undetected and no operation was performed. At the age of 71, the patient consulted an ENT specialist for a hearing problem, and, at last, the rhinolith was discovered incidentally and removed. The stone had thus remained in situ for 61 years. This case described by Bader and Hiliopoulos [22], with all its human tragedy, illustrates the fact that despite typical symptoms, the diagnosis of a rhinolith is not always easy—as Seifert noted in 1921 [23]—and this underscores the need for an endoscopic examination of the nasal cavities [24].

In most of the cases, the rhinolith is located in the inferior nasal meatus [11]. The literature also contains an occasional absolute rarity, such as a living foreign body, for example, a living leech [25]. However, the literature also contains reports of rhinoliths that were only identified because of the severe complications they caused, such as perforation of the hard palate bony destruction, and expansion of the stone into the maxillary sinus, facial tetanus, or septal perforation [5, 6, 9, 11, 26].

In the case described herein, a pronounced, nonperforated displacement of the septum to the left, together with unequivocal atrophy of the inferior and middle turbinates, was to be seen. Small rhinoliths are removed transnasally under local anaesthesia, where necessary with microscopic/endoscopic assistance. Large lesions are first fragmented within the nasal cavity, and the pieces then removed under general anaesthesia. Removal of intranasal stones with the aid of an ultrasound lithotripsy is certainly not the treatment of choice as supposed by Mink et al. [27].

## 4. Conclusion

A typical history, clinical signs, endoscopy, and radiographs showing a calcified mass point to the presence of a rhinolith. For the differential diagnosis, all possible lesions capable of blocking the nasal cavity and appearing as a calcifying mass on the X-ray must be taken into account, for example, calcifying angiofibroma, chondrosarcoma, chondroma, osteosarcoma, and calcifying polyps.

Although rhinoliths are a rare occurrence, the ENT physician should be aware of their existence.
